# Development and Evaluation of a Mobile Personalized Blood Glucose Prediction System for Patients With Gestational Diabetes Mellitus

**DOI:** 10.2196/mhealth.9236

**Published:** 2018-01-09

**Authors:** Evgenii Pustozerov, Polina Popova, Aleksandra Tkachuk, Yana Bolotko, Zafar Yuldashev, Elena Grineva

**Affiliations:** ^1^ Department of Biomedical Engineering Saint Petersburg State Electrotechnical University Saint Petersburg Russian Federation; ^2^ Institute of Endocrinology Almazov National Medical Research Centre Saint Petersburg Russian Federation; ^3^ Department of Internal Diseases and Endocrinology Pavlov First Saint Petersburg State Medical University Saint Petersburg Russian Federation

**Keywords:** blood glucose prediction, mHealth, gestational diabetes mellitus, recommender system, personalized medicine, mobile app

## Abstract

**Background:**

Personalized blood glucose (BG) prediction for diabetes patients is an important goal that is pursued by many researchers worldwide. Despite many proposals, only a few projects are dedicated to the development of complete recommender system infrastructures that incorporate BG prediction algorithms for diabetes patients. The development and implementation of such a system aided by mobile technology is of particular interest to patients with gestational diabetes mellitus (GDM), especially considering the significant importance of quickly achieving adequate BG control for these patients in a short period (ie, during pregnancy) and a typically higher acceptance rate for mobile health (mHealth) solutions for short- to midterm usage.

**Objective:**

This study was conducted with the objective of developing infrastructure comprising data processing algorithms, BG prediction models, and an appropriate mobile app for patients’ electronic record management to guide BG prediction-based personalized recommendations for patients with GDM.

**Methods:**

A mobile app for electronic diary management was developed along with data exchange and continuous BG signal processing software. Both components were coupled to obtain the necessary data for use in the personalized BG prediction system. Necessary data on meals, BG measurements, and other events were collected via the implemented mobile app and continuous glucose monitoring (CGM) system processing software. These data were used to tune and evaluate the BG prediction model, which included an algorithm for dynamic coefficients tuning. In the clinical study, 62 participants (GDM: n=49; control: n=13) took part in a 1-week monitoring trial during which they used the mobile app to track their meals and self-measurements of BG and CGM system for continuous BG monitoring. The data on 909 food intakes and corresponding postprandial BG curves as well as the set of patients’ characteristics (eg, glycated hemoglobin, body mass index [BMI], age, and lifestyle parameters) were selected as inputs for the BG prediction models.

**Results:**

The prediction results by the models for BG levels 1 hour after food intake were root mean square error=0.87 mmol/L, mean absolute error=0.69 mmol/L, and mean absolute percentage error=12.8%, which correspond to an adequate prediction accuracy for BG control decisions.

**Conclusions:**

The mobile app for the collection and processing of relevant data, appropriate software for CGM system signals processing, and BG prediction models were developed for a recommender system. The developed system may help improve BG control in patients with GDM; this will be the subject of evaluation in a subsequent study.

## Introduction

Gestational diabetes mellitus (GDM) is one of most common endocrine disorders during gestation, affecting up to 17.8% of pregnancies [1]. It is associated with short-term obstetric and perinatal complications such as preeclampsia, increased cesarean delivery rates, macrosomia, and birth injury [1], as well as long-term future metabolic health implications for the mother and the offspring (ie, increased risk of obesity and type 2 diabetes) [2]. Thus, maintaining normal blood glucose (BG) levels during pregnancy is critical for preventing adverse pregnancy outcomes and to stop the cycle that perpetuates the transmission of metabolic disease to the offspring [3].

The timeframe for effective interventions to prevent complications from GDM is usually limited to the third trimester of pregnancy and the physiology of pregnancy is rapidly changing (eg, increasing insulin resistance); therefore, women with GDM require frequent visits to health care providers to ensure good glycemic control (usually every 2-4 weeks on diet and every 1-2 weeks when treated with insulin). These frequent antenatal visits are both a considerable burden to the patients and, considering the increasing incidence of GDM, may also place significant stress on health care systems and their often-limited resources.

The technology used to remotely deliver health care—specifically, via an electronic means of communication (eg, mobile health [mHealth])—offers an appealing solution to this problem. Currently, an increasing number of articles are reporting on mobile apps for patients with different types of diabetes. Several involve mobile phone-based randomized controlled trials (RCTs) that show promising results for the self-management of diabetes [4-6].

To the best of our knowledge, no completed RCTs that assessed the effectiveness of mobile apps in GDM patients have been published, although there are several ongoing trials [7]. Approximately 70% to 85% of GDM patients can control GDM with lifestyle modification alone [8]; consequently, an effective tool for making appropriate food choices to prevent high BG levels as postprandial glucose (PPG) responses would be of particular importance for women with GDM.

The current methods for predicting PPG responses to food, albeit important in the context, are limited and imprecise. Basing predictions on meal carbohydrate content is the most common method [9], but it is not sufficiently precise in predicting PPG response [10].

Other methods of estimating PPG responses are glycemic index and glycemic load [11]. However, because both methods are based on the assessment of PPG response to the consumption of certain kinds of food, it is difficult to apply them in clinical practice when there are different food combinations and proportions [12]. Furthermore, reliable databases describing the glycemic index of different foods are absent in many countries. Moreover, several studies have found high variability in individuals’ glycemic responses to meals with identical nutritional composition [10,13], but the reasons for this variability are not yet sufficiently clear. PPG responses may depend on individual lifestyle [14], genetics [15], glucose transporter activity levels [16], and gut microbiota [10].

Zeevi et al [10] developed a machine learning algorithm that integrates multidimensional data on blood parameters, anthropometrics, physical activity, self-reported lifestyle behaviors, and gut microbiota composition to predict personalized PPG responses in healthy individuals. However, adoption of their algorithm in clinical practice may be limited by its complexity and the high cost of microbiota analyses. Further, it has not been validated on pregnant women and its usability for prediction of PPG responses during pregnancy is not known.

To the best of our knowledge, little work has been conducted on the development of PPG response prediction models for GDM patients, as the primary task for researchers remains prediction of BG levels for patients with type 1 diabetes. Further, although numerous BG prediction algorithms have been proposed, only a few projects are dedicated to the development of complete recommender system infrastructures that incorporate BG prediction algorithms for diabetes patients [17]. The development and implementation of such a system might be particularly important for patients with GDM, especially considering the high importance of BG control for these patients during pregnancy and a typically higher acceptance rate for mHealth solutions for short- to midterm usage.

Therefore, the goal of this study was to develop an infrastructure that incorporates data processing algorithms, BG prediction models, and an appropriate mobile app for patients’ electronic record management to guide BG prediction-based personalized recommendations for GDM patients.

## Methods

### Analysis

Following analysis of the literature on the available apps for diabetes management [18], main design functions for diabetes apps [19,20], app evaluations [21], and user reviews of the top diabetes apps available in the Google Play and Apple Store markets, we defined the most important points to consider within our project. For a mobile monitoring system, we derived the following core features: (1) systematic collection and persistence of patient data locally on the user device and centrally on a server, (2) data export into commonly used formats for assessment by patient and doctor, and (3) personalized recommendations based on evaluation of PPG response. Further, for the system to be sufficient for long-term monitoring, the following requirements must be satisfied: the system must be based on a commonly used architecture (in terms of devices, software, etc), the system must be sufficiently simple to use by patients without prior computer literacy, and the process of data collection and exchange should be as simple as possible.

**Figure 1:**
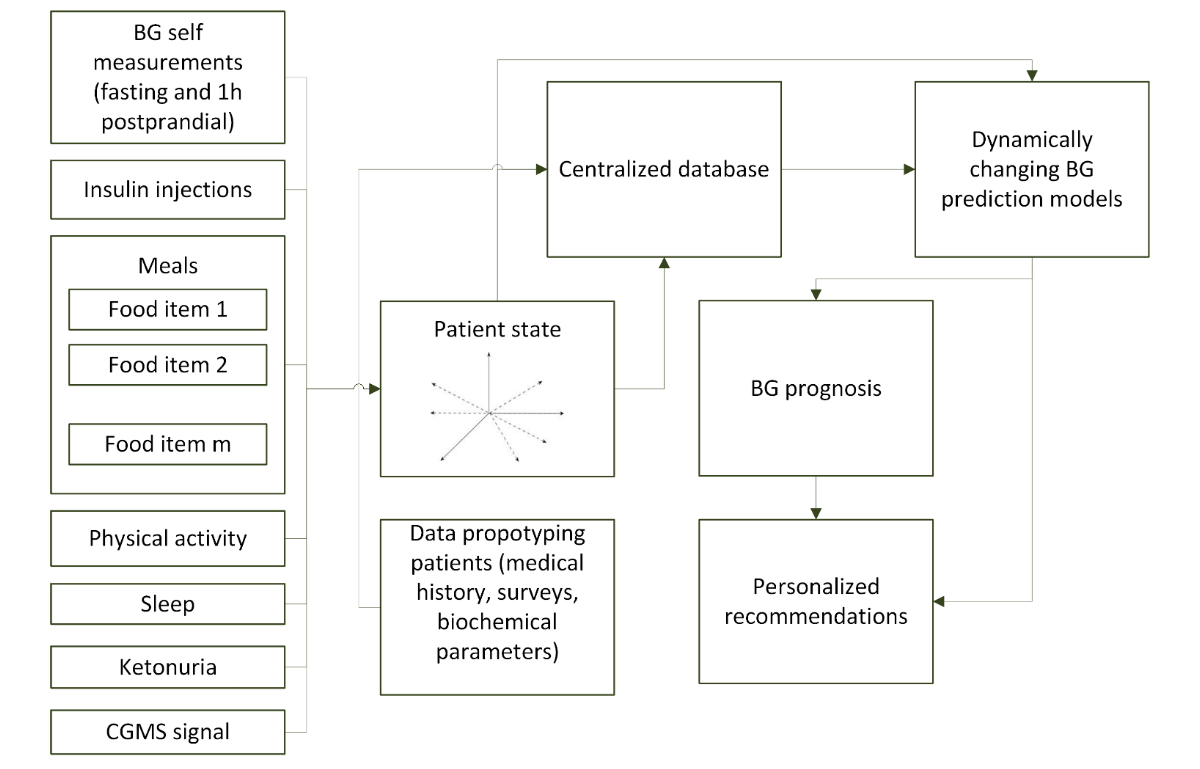
Conceptual scheme of the gestational diabetes mellitus recommender system. BG: blood glucose; CGM: continuous glucose monitoring.

The conceptual scheme of the GDM recommender system is shown in Figure 1. The core of the recommender system comprises dynamically changing BG predicting models, which are used for personalized recommendations. The architecture utilizes the data collected from different sources for all patients to constantly improve its BG predictions. The patient state is a vector in multidimensional space and contains data on preceding events (eg, meals and insulin injections) and information on BG levels collected within the continuous glucose monitoring (CGM) system signal. The patient state is combined with data prototyping patients (with the data relevant to BG regulation, such as glycated hemoglobin A_1c_ [HbA_1c_] and oral glucose tolerance test [OGTT] parameters) and the data for all patients in a centralized database that is used to train the BG prediction models. The models are dynamically retrained as new data are uploaded making their predictions more sustainable when they are used by new patients for whom only a limited amount of data has so far been recorded.

### Design

In the first step of the system design, we formulated a list of parameters for the BG predicting algorithm (Textbox 1). Because it was not feasible to track these records in a traditional paper diary and there were no solutions matching our requirements, such as apps for recording meals in a simple and hassle-free (mobile) manner and corresponding software to acquire the records of these electronic diaries alongside CGM system signals, we developed our own app to obtain the necessary records for the BG prediction algorithm.

From a technical viewpoint, the developed GDM advisory system contained the following elements: (1) a mobile app for data collection and presentation on the side of the patient, (2) a CGM system for continuous BG monitoring, and (3) a centralized server with appropriate software for data aggregation, processing, and training of BG prediction models.

**Groups and subgroups of parameters necessary for the BG prediction algorithm.**

Biomedical signals (implemented by devices)

- Continuous glucose monitoring system signal features describing postprandial glucose response

Electronic diaries records (implemented by mobile app)

- Blood glucose measurements, meals, insulin injections, physical activity, sleep duration

Individual patient characteristics

- Biometric characteristics, medical history and survey data, biochemical parameters

### Development

#### Mobile and Desktop Diary App

The mobile app was developed using the Java programming language and it supports devices running Android OS 2.3 and higher. The desktop app was also developed using the Java programming language. It supports devices running Java 7.0 and higher. The app includes a food database created based on reference books of the Russian Academy of Medical Sciences and the US Department of Agriculture (USDA) Food Composition Databases (Release 28) [22]. Complex dishes included into the database were supplemented by recipes from reference books of the Russian Academy of Medical Sciences [23].

The development process comprised several iterations and app usability was evaluated with the help of 36 GDM patients. The patients were asked to provide feedback on app usability, evaluate its core features, indicate limitations they encountered, and possible ways to improve the app. Seven major corrections were made before the study, including improving the ergonomic aspects of the app, increasing the size of the built-in food database, and enhancement of app functionality, including respective functions for modifying previously recorded data, combining existing food items in the built-in food database into new recipes, and marking particular food items as favorites. All the preceding corrections were made before commencing the study.

#### Data Processing Software

The centralized software component was based on programs that operate in an automatic sequence, evoked by a script. The complete data processing algorithm, which transforms the raw data of the electronic reports from the patients and the CGM system signals of the iPro device, comprises the following five steps.

##### Step 1

The initial signal data was recorded by the CGM system (Medtronic iPro was used in this study) and uploaded to the CareLink server used by the Medtronic iPro system, where the signals for patients were stored. For the purpose of this study, the data of the participants were retrieved from the Medtronic server in the form of CSV files by our software and processed by an automatic script.

##### Step 2

Electronic diaries, exported from the mobile app and collected on the centralized server, were processed in such a manner that every meal was stored together with the parameters characterizing previous events (eg, any meal 3 hours before the current meal and any physical exercise or sleep).

##### Step 3

**Figure 2:**
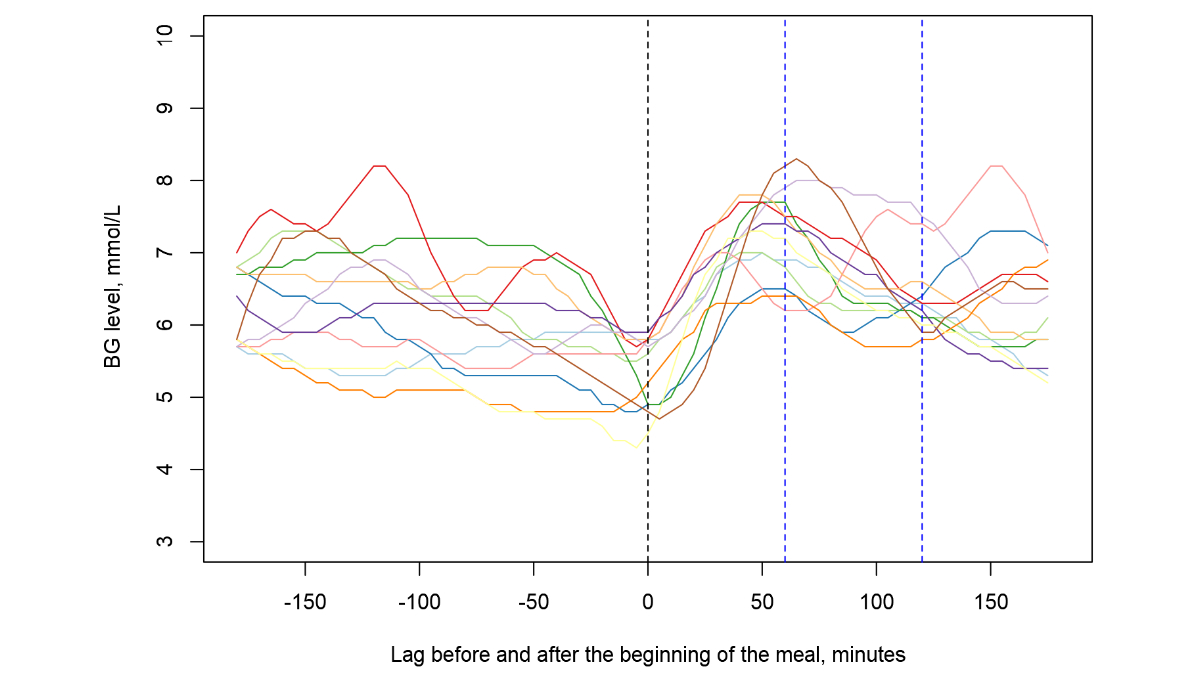
Ensemble of blood glucose (BG) curves collected 3 hours before and after meals for one of the patients. Different colors represent different meals.

Records from the electronic diaries were matched with appropriate CGM system signals for each patient: for every food intake in a diary, 3 hours of BG signals before and after the meal were collected. The information on the BG curve was stored together with food records collected in step 2. Figure 2 shows an ensemble of resulting BG curves 3 hours before and after meals, collected over the span of a week.

##### Step 4

Based on the postprandial BG curve, a set of parameters characterizing PPG response were calculated:

1. AUC60: area under the glycemic curve 1 hour after the start of the meal ([mmol/L]/hour);
2. AUC120: area under the glycemic curve 2 hours after the start of the meal ([mmol/L]/hour);
3. BG60: blood glucose level 1 hour after the start of the meal (mmol/L); and
4. Peak BG: peak value on a postprandial BG curve (mmol/L).

##### Step 5

The resulting data were supplemented by the data used to prototype the patients (medical history data, survey data, biochemical parameters). The data from different patients were combined in an integral data frame, which was the prepared input data for the BG prediction models.

#### Recruitment and Enrollment

This study was part of the ongoing clinical trial “Genetic and Epigenetic Mechanisms of Developing Gestational Diabetes Mellitus and its Effects on the Fetus” (GEM GDM; trial registration number: АААА-А16-116012210374-0), which started in July 2015. Participation in this study using the CGM system was optional for the participants of the GEM GDM trial. Pregnant women were invited to take part in this study if they were using our mobile app or our desktop app and provided accurate information concerning their food intake and BG measurements. Those who had pregestational diabetes and other diseases affecting carbohydrate metabolism were excluded. None of the participants were treated with insulin prior to or during this study. The study was approved by the ethical committee of the National Almazov Medical Research Centre, Saint-Petersburg, Russia (protocol no 119), and the participants gave their consent in writing.

In total, 66 of 158 women agreed to participate. Four women were excluded from the analysis because they provided inaccurate information on food intake during the week they wore the CGM system (see “Control of the Accuracy of Self-Reports”). As a result, 62 participants (48 pregnant women with GDM and 14 women with normal glucose tolerance) were included in the study.

The diagnosis of GDM was based on the Russian National Consensus [24] and the recommendations of the International Association of Diabetes and Pregnancy Study Groups (IADPSG) [25] based on the results of 2 hour OGTT performed during the 24th to 28th week of gestation. Pregnant women without diabetes were included as controls.

#### Measures

Glucose was measured for 7 days using the iPro2 CGM with Enlite sensors (Medtronic, Minneapolis, MN, USA) and independently calibrated with the Accu-Check Performa Nano blood glucose meter (Roche Diabetes Care, Indianapolis, IN, USA) for a minimum of four measurements per day.

During that week, participants were instructed to record all daily activities, including meals with exact components and weights, using our mobile app or our desktop app.

Prior to the study, the women were questioned about their clinical characteristics and completed a special questionnaire under supervision. The questionnaire consisted of the following sections: frequency of consumption of basic products in a week, physical activity, and smoking before and during pregnancy. The sections of the form were defined in a semiquantitative manner, reflecting different frequency levels of consuming certain products and performing physical activity (low, medium, and high). The description of these semiquantitative variables is presented in Multimedia Appendix 1. This questionnaire was previously reported [26].

#### Control of the Accuracy of Self-Reports

To avoid biases resulting from inaccurate self-reports about daily activities, especially meals, we took several precautions. The women were provided with kitchen scales to measure the weight of each kind of food consumed at home (in grams) and were asked to check the weight of meal components consumed in restaurants and other public catering places. The reports resulting from the collection of data on food intake were analyzed by endocrinologists and discussed with the participants in detail. If the BG curves collected via the iPro2 CGM showed that two or more food intakes per week were not documented in the app or that two or more BG measurements checked in the glucometer memory differed from the BG levels reported by a participant, the data were excluded from the analysis (four women).

#### Statistical Analysis

Data were statistically processed with SPSS 22.0 [27], MATLAB 2016r [28], R 3.4.0 [29], and Python 2.7.14 [30]. The data are presented as the mean and standard deviation. Differences in the quantitative characteristics of the groups were assessed with Student *t* test. The chi-square criterion was used to compare the distribution of qualitative characteristics. The differences were considered significant at *P*<.05.

#### Blood Glucose Prediction Model

After the data processing phase, the data were used to create the BG prediction model. We developed linear regression models with the use of lasso regularization for feature selection and coefficient tuning [31] to avoid overfitting. Linear regression was chosen because of its good interpretability, simplicity, rapid tuning, and adequate accuracy in comparison with other methods performing the task.

Participants were separated into training and test sets in the proportion of 80%:20% and 20-fold cross-validation was used for parameter tuning.

The optimization task for lasso regularization was ║y–Xω║^2^+λ║ω║→min_ω_, λ≥0, where *y* is a vector of output values, *ω* is a vector of weights, *X=[x*_1_*,...,x*_n_*]* is a set of input values for all objects in the dataset, and *λ* is the tunable regularization coefficient (double vertical bars stand for a norm of a vector/matrix).

As a dependent indicator, the features of PPG response (AUC60, AUC120, BG60, and peak BG) were determined for appropriate models. The following parameters were imputed in the dataset as potential predictors of the features of PPG response:

1. Anthropometric parameters (eg, age, weight, body mass index [BMI], gestational age, and systolic and diastolic blood pressure),
2. Medical history data (impaired glucose tolerance; polycystic ovary syndrome; family history of diabetes; number of pregnancies, abortions, deliveries, and miscarriages; arterial hypertension; the use of combined oral contraceptive pills before pregnancy; and GDM in history), biochemical parameters (fasting, 1-hour and 2-hour BG levels at OGTT, fasting insulin, HbA_1c_, total cholesterol level, very low density and high density lipoproteins, and triglycerides at the time of OGTT).
3. Survey data: 11 parameters associated with the consumption of certain product groups (fruits, pastries, skimmed dairy products, legumes, meat, sausage products, dried fruits, fish, whole grain bread, sauces, and vegetables), three parameters related to beverages (alcohol, sweet drinks, and coffee), and three parameters characterizing physical activity (walking, climbing the stairs, and performing sports). For each listed parameter, the intensity was estimated on an ordinal scale of three levels: low, medium, and high. Smoking was marked as “yes” or “no.” Smoking, alcohol intake, and physical activity parameters were assessed separately before and during pregnancy. Because none of the participants reported the highest category of activity for frequency of sports activities (>3 days/week), frequency of climbing the stairs (>16 flights/day), and legume consumption (>2 portions/week) at the time of glucose self-monitoring, we recorded the remaining two categories into binary variants (“yes”: medium category; “no”: the lowest category of activity) for statistical analyses. Walking duration and coffee consumption were coded as variables with three levels: (0 for low, 1 for medium, and 2 for high).
4. Current and preceding meal: 33 parameters including type of food intake (1=breakfast, 2=lunch, 3=dinner, 4=snack); macronutrient and micronutrient content (water [g], energetic value [kcal], fats [g], carbohydrates [g], dietary fibers [g], sugars [g], calcium [mg], iron [mg], phosphorus [mg], zinc [mg], copper [mg], vitamin С [mg], riboflavin [mg], niacin [mg], thiamin [mg], vitamin B6 [mg], folate [mcg], folic acid [mcg], retinol [mcg], retinol equivalent [mcg], alpha-carotene [mcg], beta-carotene [mcg], vitamin E [mg], vitamin D [mg]); the presence of a preceding meal within 3 hours before the index meal (yes/no); and the amount and percentage of carbohydrates in the preceding meal.

The coefficients were tuned via a coordinate descent optimization algorithm (using the glmnet package for R) [32]. The λ coefficient for each model was chosen in such a manner as to obtain the smallest number of nonzero coefficients, at which the mean squared error, estimated during cross-validation, was in the range of one standard deviation from the best model fit. This allowed us to obtain a simple, yet sufficiently accurate model.

## Results

### Mobile App

The mobile app for data collection and exchange was developed for the Android OS. A desktop app with the same functionality was also developed for users not in possession of an Android device (both are referred to simply as “app”).

The app contains 18 different screens, including the main menu, user input forms, record management and information, user settings, data export form, and help. Some of these screens are presented in the animation included in Multimedia Appendix 2.

The SQLite database in the app consisted of 13 tables (tables for records on BG, insulin, physical activity, sleep, ketones, meals, and meal items; tables with built-in and user food databases and user data) containing the data for different types of user records as well as the built-in food database. The built-in food database, collected from open sources (including the Scientific Research Institute of Nutrition of the Russian Academy of Medical Sciences and the USDA food databases), made it possible to track 27 food parameters (macronutrients and micronutrients) without patient input, because involving the patients could lead to mistakes and additional burden from the patients’ perspective.

**Figure 3:**
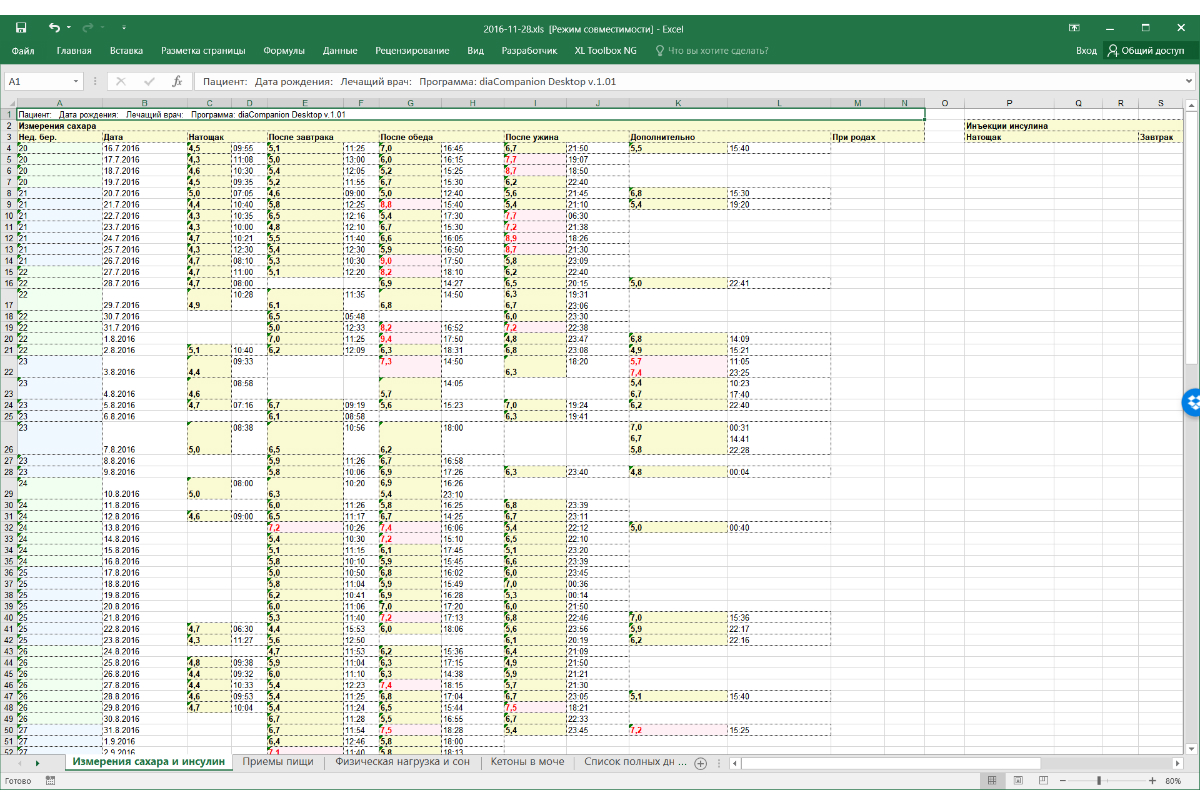
Example of a standardized report exported from the app.

The app allowed the users to export data in Excel spreadsheets and store them on their devices as well as to send them remotely to physicians (Figure 3).

### The Data Processing Algorithm

**Figure 4:**
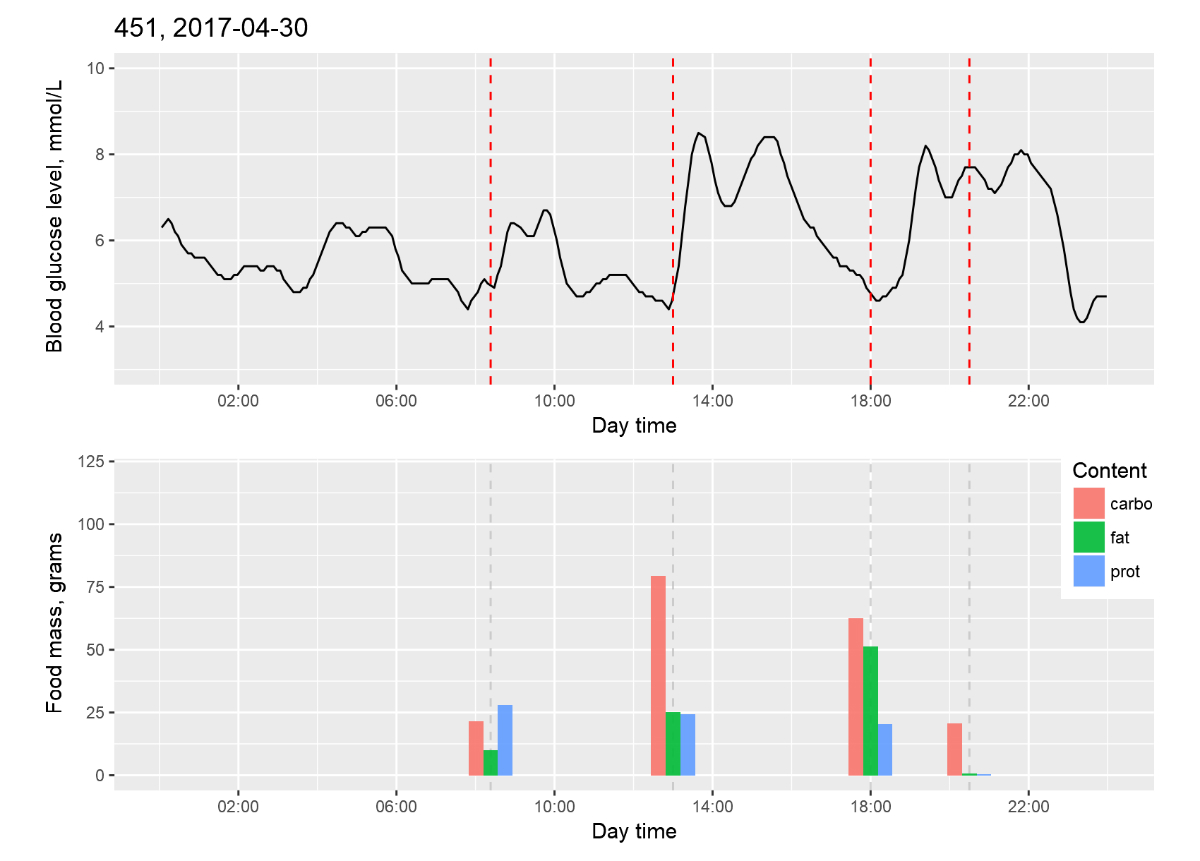
Result of data matching between the continuous glucose monitoring system signal and the electronic diary.

Using the preceding methods, an algorithm transformed the data for the amount and kind of consumed food, the start time of food intake, physical activity, duration of sleep, and current BG level (received from the CGM system) into a BG prediction parameter used to establish a recommender system. An example of the results of data matching between the CGM system signal and the electronic diary is shown in Figure 4 and more examples are shown in Multimedia Appendix 3.

### Participants’ Characteristics

The main characteristics of the participants are presented in Table 1. The women with GDM had higher BMI and higher levels of HbA_1c_ and plasma glucose (PG) during OGTT than the controls.

**Table 1:** Characteristics of the participants (N=62).

Characteristic	GDM (n=48)	Control (n=14)	*P* (two-sided test)
Age (years), mean (SD^a^)	32.1 (4.0)	29.8 (2.9)	.06
Prepregnancy BMI^b^ (kg/m^2^), mean (SD)	26.4 (6.4)	21.1 (3.4)	.006
HbA_1c_^c^ (%), mean (SD)	5.13 (0.40)	4.84 (0.40)	.03
Gestational age (weeks), mean (SD)	31.4 (3.0)	31.4 (2.8)	>.99
BP^d^ systolic (mm Hg), mean (SD)	118.9 (10.6)	117.5 (13.9)	.70
BP diastolic (mm Hg), mean (SD)	75.1 (7.9)	74.8 (14.9)	.90
Arterial hypertension, n (%)	20 (42)	5 (36)	.77
OGTT^e^ fasting PG^f^ (mmol/L), mean (SD)	5.0 (0.7)	4.2 (0.5)	<.001
OGTT 1-h PG (mmol/L), mean (SD)	9.6 (2.3)	6.3 (1.6)	<.001
OGTT 2-h PG (mmol/L), mean (SD)	8.4 (2.4)	5.5 (1.4)	<.001
Fasting serum insulin (pmol/L), mean (SD)	96.4 (52.8)	93.9 (81.9)	.91

^a^SD: standard deviation.

^b^BMI: body mass index.

^c^HbA_1c_: glycated hemoglobin A_1c_.

^d^BP: blood pressure.

^e^OGTT: oral glucose tolerance test.

^f^PG: plasma glucose.

**Table 2:** Glycemic response and meal characteristics for gestational diabetes mellitus (GDM) and control patients.

Characteristic	GDM, mean (SD^a^)	Control, mean (SD)	*P* (two-sided test)
Fasting BG^b^ (mmol/L)	5.1 (0.7)	5.0 (0.6)	<.001
BG60^c^ (mmol/L)	6.2 (1.0)	5.9 (0.9)	<.001
AUC60^d^ (mmol/L*hour)	5.77 (0.80)	5.62 (0.74)	.02
AUC120^e^ (mmol/L*hour)	5.86 (0.78)	5.68 (0.70)	<.001
BG Rise 1h after meal (mmol/L)	1.5 (1.0)	1.6 (1.0)	.66
Postprandial peak BG (mmol/L)	6.6 (1.0)	6.5 (1.0)	.02
Time to peak BG (minutes)	75.0 (43.7)	73.6 (46.0)	.68
Carbohydrates per meal (g)	31.8 (22.2)	51.5 (31.5)	<.001
Proteins per meal (g)	22.5 (15.1)	22.9 (15.7)	.72
Fats per meal (g)	19.6 (15.1)	25.2 (17.0)	<.001
Energy per meal (kcal)	398 (209)	530 (279)	<.001

^a^SD: standard deviation.

^b^BP: blood pressure.

^c^BG60: blood glucose level 60 minutes after the meal.

^d^AUC60: area under the postprandial blood glucose curve 60 minutes after the meal

^e^AUC120: area under the postprandial blood glucose curve 120 minutes after the meal.

### Characteristics of Meals and Glycemic Responses

The average characteristics of meals and glycemic responses are presented in Table 2. Patients with GDM consumed significantly lower amounts of carbohydrates and fats in their meals; therefore, the energy content of their meals was considerably lower than that of the control group. Fasting and postprandial BG levels were significantly higher in patients with GDM than in those in the control group, whereas the actual rise in BG level after meals did not vary significantly in these groups owing to lower average carbohydrate consumption in the GDM group. The area under the curve (AUC) for BG level 1 and 2 hours after the beginning of the meal was also larger for patients in the GDM group, even considering the lower average carbohydrate consumption in this group.

### Blood Glucose Prediction Models

The significant model coefficients are presented in Table 3. The BG level 2 hours after OGTT was a heightening factor for all the variables describing glycemic response, corresponding to worse BG regulation in participants with GDM. Some of the parameters describing lifestyle during pregnancy were significant in predicting PPG response: reported physical activity was a lowering factor for all the variables describing glycemic response, where they were found to be significant, as well as reported high consumption of legumes, although high consumption of coffee appeared to be a heightening factor.

Despite their simplicity, the developed linear regression models proved to be highly efficient in their prediction of the PPG response feature. Model performance was estimated using standardized metrics. The correlation between real and predicted values (*R*), root mean square error (RMSE), mean absolute error (MAE), and mean absolute percentage error (MAPE) were estimated for each of the proposed models (Table 4). The resulting table shows only marginally worse results for the test set, which may be due to overfitting. When comparing the MAPE of the presented models, it might be considered that the models predicting AUC on a postprandial curve perform better.

**Table 3:** Coefficients of the linear regression models predicting different features of postprandial glucose (PPG) response.

Parameter	AUC60^a^	AUC120^b^	BG60^c^	Peak BG^d^
Intercept	1.6246	2.5650	2.1860	3.4590
1. BG level before meal (mmol/L)	0.6877	0.6033	0.4116	0.5959
2. Breakfast (yes/no)	0.2927	0.2337	0.2746	0.2832
3. Carbohydrates (g)	0.0030	0.0034	0.0072	0.0093
4. Starch (g)	—	0.0017	—	0.0024
5. Carbohydrates (%)	0.1951	0.0289	0.0902	—
6. Proteins (%)	—	—	—	–0.4503
7. Preceding meal (yes/no)	—	–0.0539	–0.1570	–0.0730
8. Carbohydrates in preceding meal (g)	—	—	—	–0.0029
9. OGTT^e^ fasting BG (mmol/L)	—	—	0.2974	—
10. OGTT 2h BG (mmol/L)	0.0484	0.0397	0.0356	0.1036
11. Fasting serum insulin (pmol/L)^f^	—	—	—	0,0021
12. Sports (≥2 days/week, yes/no)^g^	–0.1416	—	—	—
13. Climbing stairs (≥4 flights/day, yes/no)^g^	–0.0497	–0.1938	–0.1860	–0.0364
14. Walking (≤30, 31-60, ≥61 min/day for 0, 1, 2)^g^	—	–0.1062	–0.0864	–0.3349
15. Legumes >1/week ( (yes/no)^g^	—	—	—	–0.2184
16. Coffee (0-1, 2-3, >3 cups/day for 0, 1, 2)^g^	0.0025	0.1173	0.0738	0.0311

^a^AUC60: area under the postprandial blood glucose curve 60 minutes after the meal.

^b^AUC120: area under the postprandial blood glucose curve 120 minutes after the meal.

^c^BG60: blood glucose level 60 minutes after the meal.

^d^BP: blood pressure. Peak BG: peak BG level on a 3-hour postprandial BG curve.

^e^OGTT: oral glucose tolerance test.

^f^Measured at the day of OGTT.

^g^During pregnancy.

**Table 4:** Estimation of model performance.

Characteristic and set		*R*	Root mean square error	Mean absolute error	Mean absolute percentage error
**AUC60**^a^					
	Test	.79	0.62	0.52	9.3%
	Training	.78	0.51	0.40	6.8%
**AUC120**^b^					
	Test	.75	0.61	0.48	9.1%
	Training	.75	0.51	0.39	6.6%
**BG60**^c^					
	Test	.69	0.81	0.66	12.0%
	Training	.66	0.75	0.56	8.9%
**Peak BG**^d^					
	Test	.48	1.00	0.77	12.2%
	Training	.74	0.68	0.53	8.0%

^a^AUC60: area under the postprandial blood glucose curve 60 minutes after the meal.

^b^AUC120: area under the postprandial blood glucose curve 120 minutes after the meal.

^c^BG60: blood glucose level 60 minutes after the meal.

^d^BP: blood pressure. Peak BG: peak BG level on a 3-hour postprandial BG curve.

## Discussion

### Principal Results

Our infrastructure, including the mobile app for patients’ electronic record management and data processing algorithms for matching of the CGM system signal and electronic diary, enabled the collection and analysis of data on 909 food intakes and corresponding postprandial BG curves from 62 pregnant women. Combining these data with patients’ characteristics (eg, HbA_1c_, BMI, age, and lifestyle parameters) facilitated the development of models that accurately predict PPG response for real-life meals and can be implemented in our app for personalized prediction of PPG response and subsequent decision-making support.

To the best of our knowledge, no previous study has developed a tool for personalized prediction of PPG response in pregnant women. As the physiology of pregnancy differs significantly from that of the nonpregnant state, models created on nonpregnant participants have no potential to be applied to the management of pregnant women.

A unique feature of our app is the ability to integrate food choices based on our PPG response models in a decision-making algorithm. This will enable the app to give personalized advice concerning each upcoming meal in order to achieve desired BG levels. To adapt the personalized model to her specific requirements, a woman simply needs to fill in a short questionnaire when the app is first started (the questionnaire covers parameters 9-16 listed in Table 3). In order to receive on-site personalized nutrition advice, participants have to enter the desired food components with exact weights before meals. The app will calculate the predicted PPG response and, in case the recommended levels are exceeded, will suggest how to reduce the carbohydrate content of the desired meal by reducing the amount of carbohydrate-rich products or by suggesting variants of products for replacement.

The significant potential of our app lies in the ability to capture data, provide decision support, and share data with health care providers, thus promoting communication. Although the efficacy in terms of use of our app is yet to be tested in an RCT, we believe that it already has potential for supplementing traditional care, especially between visits to the clinic, when patients can be provided with on-site personalized recommendations and education. Our app may also be helpful for the collection of large datasets for future statistical analyses.

For short-term use (eg, pregnancy), mHealth solutions might be more widely accepted and there will potentially be less app attrition compared to patients with chronic diseases.

To test and train PPG response prediction models, we used data of women with normal glucose tolerance in line with the data from GDM patients. We consider this approach appropriate because the postprandial BG levels of women with normal glucose tolerance and GDM diagnosed under the new IADPSG criteria often overlap, as shown in Table 2. Because women with normal glucose tolerance are not required to adhere to a diet, they consume much more carbohydrates. Consequently, they often have BG levels similar to GDM patients, thus enabling us to assess the impact on PPG response of products with a more variable carbohydrate content.

Our approach of making patients select foodstuff from a prebuilt database is a good way to standardize and ease the procedure of food tracking as well as to increase data reliability. On the other hand, this created an additional problem for patients who had to search for their desired food items.

We provided patients with the possibility to create their own food items by combining existing items, but only a few people found this feature useful. There are two main reasons why we refrained from allowing users to add food items to the app database on their own. Firstly, there is virtually no way to find a comprehensive list of all the nutritional characteristics we would like to track (previous 25 features) for potential food items. Secondly, there is a concern with the quality of data available on publicly available Internet sources. Allowing users to add new items into the database might lead to mistakes and incomplete data, which might in turn cause bias in the statistical analyses.

The most important predictors of PPG response that remained significant for all models were BG level before meal, quantity of carbohydrates in the meal, type of food (breakfast was a heightening factor), 2-hour BG level at OGTT, frequency of coffee consumption as a heightening factor, and the level of physical activity expressed as climbing stairs during pregnancy, which lowered PPG response. All these parameters are expected with regard to physiology and are in line with the results of previous studies addressing factors determining glucose metabolism [33-35].

### Comparison With Prior Work

Table 5 compares the prediction quality of the developed predicting models to that of BG predicting models of different types presented in the literature. All models exhibit adequate accuracy that allows them to be used for patient assistance. The predictive power of the developed models and those presented in recent scientific papers is of equal quality in terms of *R*, RMSE, MAE, and MAPE. Considering that the developed model is simple, interpretable, and requires little time to tune its parameters, in which it fulfills the requirement for dynamical coefficient updates, the model must be stated as adequate for the pursued goal.

### Limitations

Our study has several limitations. Because the data on food intake were self-reported by participants through the app, there is the potential for biases due to inaccurate reports. For example, women who are overweight or gain excessive weight during pregnancy often underestimate their actual consumption of foods that are considered harmful. This is a typical drawback of any epidemiological study assessing nutrition. However, we used hints found in the data to reduce this kind of bias, as described previously (see Control of the Accuracy of Self-Reports section).

In addition, the PPG response prediction models are based on data from a relatively small sample size. However, the resulting PPG response prediction models have predictive power of equal quality to those presented in recent scientific papers [10,36-39]. Overall, we believe that the developed PPG response prediction models are sufficiently accurate to form the basis for a subsequent self-management intervention.

There are several concerns limiting the current use of the developed app with the built-in decision-making algorithm. Our app is so far only available for Android devices and PCs; unfortunately, this prevents iPhone users from accessing the app using their mobile phone (they can access it only using their desktop computer). We plan to increase the number of potential users by implementing an app for iPhone users as well. We are also considering implementing a responsive Web app to allow usage of a wide range of platforms and form factors.

This study did not include insulin treatment. Thus, the current models are not designed to predict PPG response after insulin injections. This will need to be evaluated in the future.

**Table 5:** Comparison between the proposed model and prior developed models.

Value and author(s)		Mathematical model	*R*	Root mean square error (mmol/L*hour; mmol/L)	Mean absolute percentage error (%)
**AUC120**^a^					
	Pustozerov et al^b^	Linear regression	.71	0.62	6.8
	Zeevi et al [10]	Boosted decision trees	.70	—	—
**BG60**^c^					
	Pustozerov et al^b^	Linear regression	.69	0.82	12.0
	Plis et al [36]	Support vector regression	—	1.97	—
	Wang et al [37]	Autoregression, support vector machines, and neural network	—	0.53-1.29	5.1-16.6
	Perez-Gandia et al [38]	Neural network	—	1.38-1.60	—
	Perez-Gandia et al [38]	Autoregression	—	1.67-2.17	—
	Stahl [39]	Lehmann and Deutsch, Dalla Man	—	1.24-1.73	—

^a^AUC60: area under the postprandial blood glucose curve 60 minutes after the meal.

^b^Our model.

^c^BG60: blood glucose level 60 minutes after the meal.

Our app currently lacks the ability to wirelessly upload BG readings. We also plan to overcome this inconvenience in the future.

The algorithm used for data processing is currently only semiautomatic and requires the intervention of the researcher in the final data processing steps. In the future, all the system software should conceivably be rewritten in a high-level programming language.

The developed PPG response prediction models are based and validated on the data of women in the third trimester of pregnancy. Therefore, it should not be used in the first and second trimesters. However, GDM is usually diagnosed in the third trimester and, according to the recommendations of some diabetes associations, can be diagnosed only in the third trimester [8]. Because Russian guidelines suggest diagnosing GDM at any time during pregnancy [24], we are planning an additional study to validate the PPG response prediction models for the first and second trimesters.

To improve BG control, as well as maternal and fetal outcomes, the efficacy of this app for the management of women with GDM needs confirmation in an RCT and we plan to perform such an RCT.

### Conclusions

This infrastructure comprising the data processing algorithms, the BG prediction models, and the mobile app for patients’ electronic record management can be useful for guiding BG prediction-based personalized recommendations for GDM patients. The accuracy of the prediction models was validated on training sets of patients with 20-fold cross-validation for parameters tuning. The efficacy of the implementation in terms of providing health care to women with GDM to reduce BG levels and pregnancy complications will be evaluated in a future RCT.

## Appendix

Multimedia Appendix 1 Lifestyle questionnaire.

Multimedia Appendix 2 Mobile app forms.

Multimedia Appendix 3 CGMS and electronic diary data merging.

## Abbreviations

AUC: area under the curve
AUC60: area under the postprandial blood glucose curve 60 minutes after the meal
AUC120: area under the postprandial blood glucose curve 120 minutes after the meal
BG: blood glucose
BG60: postprandial blood glucose 60 minutes after the meal
BMI: body mass index
CGM: continuous glucose monitoring
GDM: gestational diabetes mellitus
HbA_1c_: glycated hemoglobin A_1c_
MAE: mean absolute error
MAPE: mean absolute percentage error
mHealth: mobile health
OGTT: oral glucose tolerance test
Peak BG: peak blood glucose value on a 3-hour postprandial blood glucose
PG: plasma glucose
PPG: postprandial glucose
RCT: randomized controlled trial
RMSE: root mean square error
