# Supplementary material for: Development and Evaluation of a Mobile Personalized Blood Glucose Prediction System for Patients With Gestational Diabetes Mellitus
Source: JMIR Mhealth Uhealth. 2018 Jan 9;6(1):e6. doi: 10.2196/mhealth.9236 (PMC5780619; doi:10.2196/mhealth.9236)
Supplement: Multimedia Appendix 1 [file mhealth_v6i1e6_app1.pdf]

	Score		
	0	1	2
Physical activity			
Light walking, min/day	<30	30–60	>60
Climbing the stairs, no. floors/day	<4	4–16	>16
Sports (at least 30 min of more than moderate intensity), days/week	<2	2–3	>3
Servings per wek			
Fruits	<6	6–12	>12
Pastries	<2	2-4	> 4
Skimmed dairy products	< 3	3-6	> 6
Legumes	<1	1-2	> 2
Red and processed meats	<3	3-6	> 6
Dried fruits and nuts	0	1-3	> 3
Fish	<3	3-6	> 6
Whole-wheat bread	< 1	1-3	> 3
Sauces	< 2	1-4	> 4
Vegetables and salads	<6	6–12	>12
Sausages, <1/week, 1–3/week, >3/week	< 1	1-3	> 3
Alcohol	< 1	1-3	> 3
Sweet beverages	<2	2-4	> 4
Coffee	0-1	2-3	> 3
Smoking			
Before pregnancy	no	yes	
During pregnancy	no	yes	
